# From Cryptic Toward Canonical Pre-mRNA Splicing in Pompe Disease: a Pipeline for the Development of Antisense Oligonucleotides

**DOI:** 10.1038/mtna.2016.75

**Published:** 2016-09-13

**Authors:** Atze J Bergsma, Stijn LM in ‘t Groen, Frans W Verheijen, Ans T van der Ploeg, WWM Pim Pijnappel

**Affiliations:** 1 Department of Clinical Genetics, Molecular Stem Cell Biology, Erasmus Medical Center, Rotterdam, The Netherlands; 2 Department of Pediatrics, Erasmus Medical Center, Rotterdam, The Netherlands; 3 Center for Lysosomal and Metabolic Diseases, Erasmus Medical Center, Rotterdam, The Netherlands; 4 Department of Clinical Genetics, Molecular Diagnostics, Erasmus Medical Center, Rotterdam, The Netherlands

**Keywords:** cryptic splice site, morpholino antisense oligonucleotides, muscle, Pompe disease, splicing correction

## Abstract

While 9% of human pathogenic variants have an established effect on pre-mRNA splicing, it is suspected that an additional 20% of otherwise classified variants also affect splicing. Aberrant splicing includes disruption of splice sites or regulatory elements, or creation or strengthening of cryptic splice sites. For the majority of variants, it is poorly understood to what extent and how these may affect splicing. We have identified cryptic splicing in an unbiased manner. Three types of cryptic splicing were analyzed in the context of pathogenic variants in the acid α-glucosidase gene causing Pompe disease. These involved newly formed deep intronic or exonic cryptic splice sites, and a natural cryptic splice that was utilized due to weakening of a canonical splice site. Antisense oligonucleotides that targeted the identified cryptic splice sites repressed cryptic splicing at the expense of canonical splicing in all three cases, as shown by reverse-transcriptase-quantitative polymerase chain reaction analysis and by enhancement of acid α-glucosidase enzymatic activity. This argues for a competition model for available splice sites, including intact or weakened canonical sites and natural or newly formed cryptic sites. The pipeline described here can detect cryptic splicing and correct canonical splicing using antisense oligonucleotides to restore the gene defect.

## Introduction

The Human Gene Mutation Database, with >7,000 monogenic disorders, reports that ~9% of all known disease causing variants (numbering >170,000) are located at or near splice sites (http://www.hgmd.cf.ac.uk/).^1^ In addition, it has been estimated that an additional ~20% of pathogenic variants affect splicing but are located at more distant locations in introns or in exons.^2,3,4^ Consequences of aberrant splicing include exon skipping, intron retention, cassette exon inclusion, and use of cryptic splice sites.^5,6,7^ There are several ways in which cryptic splicing may be induced by a pathogenic variant. One possibility is the generation of a new intronic splice donor or acceptor site that outcompete a canonical splice site. Alternatively, a natural cryptic splice site may be present that is normally suppressed by a canonical splice site, but becomes the dominant splice site due to weakening of the canonical splice site by a pathogenic variant. Weakening of a canonical splice site can be a consequence of pathogenic variants that disrupt conventional splicing elements, including the splice site itself,^8^ the polypyrimidine tract,^9^ branch point sequence,^10^ or variants that modulate the activity of exonic and intronic splicing silencer or enhancer elements^11,12^ (reviewed by Scotti *et al.*^5^). Another mechanism represents formation of a newly formed cryptic splice site at an exonic rather than intronic location. The location of a cryptic splice site in coding or noncoding RNA is likely important because exons and introns are defined within the pre-mRNA via interaction with distinct RNA binding proteins that may differentially affect cryptic splice site utilization. These include members of the SR and hnRNP families.^13,14^ However, for many disorders and gene variants, those that may affect splicing are poorly characterized.

Pre-mRNA splicing is regulated at several levels. Splice sites have short consensus sequences that are recognized by the spliceosome complex members, while additional, poorly defined sequences present in the introns and exons can modulate splice site choice. In addition, other mechanisms can affect splicing including the speed of RNA pol II transcription, chromatin structure, histone modifications, alternative transcription start and termination sites, GC content, mRNA export, mRNA stability, and expression levels of critical splicing proteins.^13,14^ These are likely reasons why *in silico* prediction of splice site choice is difficult and experimental testing is required to elucidate the effect of pathogenic gene variants.

We have previously described a splicing assay that can be used for the detection and quantification of aberrant splicing in Pompe disease (OMIM232300).^15^ It is based on the analysis of all exons of the *acid α-glucosidase* (*GAA*) gene. Besides the detection of aberrant splicing that may be linked to the disease, this approach provides quantitative information on the extent of leaky wild type splicing, which is informative for disease severity, and on the splicing mechanism, which is required to design methods for splicing correction. Antisense oligonucleotides (AONs) provide a way to interfere with splicing in a sequence-specific manner.^16^ AONs can be targeted to the pre-mRNA region of interest and they can repress the activity of a splicing motif. The clinically most advanced examples are enhancement of exon inclusion of *survival of motor neuron 2 (SMN2)* pre-mRNA in spinal muscular atrophy,^17^ and exon skipping of the *dystrophin* pre-mRNA in Duchenne muscular dystrophy (DMD).^18^ Other preclinical examples include modulation of splicing in Hutchinson-Gilford progeria syndrome^19^ and type I Usher syndrome.^20^ The developments for enhancing cellular uptake using conjugation of AONs to cell penetrating peptides^21,22,23^ and via coadministration of hexose^24^ is expected to further stimulate the clinical development of AON-based splicing modulation.

Here, we present a pipeline in which characterization of aberrant splicing in Pompe disease is first performed using a generic splicing assay.^15^ The information obtained is then used to design an AON based on inhibition of cryptic splicing, and the AON is tested in patient-derived fibroblasts. Pompe disease is an autosomal recessive disorder caused by variants in the *GAA* gene and results in lysosomal glycogen accumulation that predominantly affects skeletal muscle in the childhood/adult onset form of the disease.^25^ Currently, enzyme replacement therapy is available, but there are several reasons to develop alternative therapies, including the heterogenic clinical response, the inability to completely counteract the disease, and the extremely high costs.^26,27,28,29^ Three pathogenic *GAA* variants with different effects on cryptic splicing were analyzed, and this enabled the successful design of AONs that promoted splicing correction. The pipeline from splicing analysis to AON-based splicing correction may provide a basis for personalized medicine in human disease.

## Results

### Unbiased splicing analysis of all exons identifies aberrant splicing from an unknown *GAA* allele in patient 1

**Figure 1:**
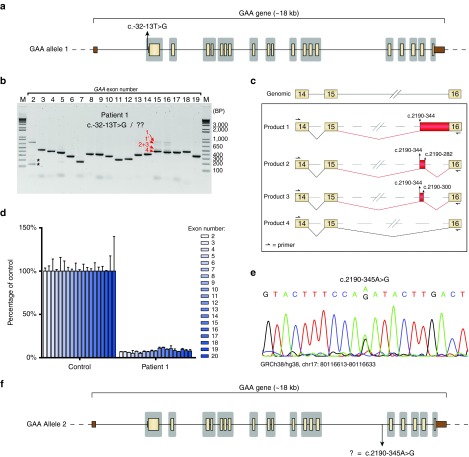
**Identification of a deep intronic pathogenic variant in patient 1 using the splicing assay.** (**a**) Scaled cartoon of *acid α-glucosidase *(*GAA*) allele 1 from patient 1 carrying the c.-32-13T>G (IVS1) variant. Coding exons are indicated in yellow, untranslated regions in brown. Lines represent introns. Areas in gray indicate the regions that are sequenced as part of standard diagnostic practice. (**b**) Flanking exon reverse-transcriptase-polymerase chain reaction (RT-PCR) of all *GAA* coding exons. Asterisks indicate known aberrant splicing products caused by the IVS1 variant. Products 1–4 indicate novel mRNA products. Product 1′ refers to a secondary structural variant of product 1 (see **Supplementary Figure S1b**). (**c**) Cartoon of splicing products 1–4. Boxes indicate exonic regions, (dashed) lines represent intronic regions. Aberrant splicing events are indicated in red. (**d**) Exon internal RT-quantitative PCR analysis of *GAA* exons 2–20. Data are normalized for *β-actin* and for a healthy control. Data represent means of three technical replicates ± SD. (**e**) Sequence analysis of genomic DNA of the region surrounding the c.2190–344 cryptic splice acceptor site. (**f**) Cartoon of *GAA* allele 2 showing the location of the c.2190-345A>G variant.

Patient 1 was diagnosed with Pompe disease based on a deficiency of GAA enzymatic activity in fibroblasts and leukocytes (**Table 1**). Standard diagnostic DNA analysis, which includes Sanger sequencing of exons and short flanking intronic regions, identified the c.-32-13T>G (IVS1) *GAA* variant on one allele,^9^ but the pathogenic variant on the second allele was not identified (**Figure 1a**). We then applied our splicing assay (ref. 15) to test whether the unknown variant may be a regulatory or deep intronic splicing variant. Flanking exon polymerase chain reaction (PCR) analysis of all spliced *GAA* exons showed the expected aberrant splice products for exon 2 caused by the IVS1 variant, including a full exon 2 skip, a partial exon 2 skip, and leaky wild type splicing (**Figure 1b**).^15,30,31^ Amplification of exons 15 and 16 revealed several higher molecular weight PCR products of low abundance that were not normally observed in cells from healthy controls.^15^ To identify these, the exon 15 PCR products were directly processed by Topo cloning, and 93 clones were analyzed by Sanger sequencing. Four mRNAs were identified (**Figure 1c** and **Supplementary Figure S1a**). All aberrantly spliced products (1–3) utilized a cryptic splice acceptor in intron 15 at c.2190–344, and showed inclusion of various parts of intron 15. Product 1 included the downstream intronic region up to the splice acceptor of exon 16. This product contains a premature stop codon and is likely to undergo mRNA decay, in agreement with the low abundance of this product. Product 2 and 3 included a small part of intron 15 by using the cryptic splice donor sites at c.2190–282 and c.2190–300, respectively. Product 2 contained the same premature stop codon as product 1, and was likely degraded as well. In product 3, the reading frame remained intact. Therefore, the low abundance of the PCR product suggests minimal usage of the c.2190–300 splice site. Product 4 contained the canonical splice junctions and was the most abundant product of exon 15 amplification. It should be noted that the canonically spliced exon 15 product is also expressed from the IVS1 allele. No unique sequence of product 1′ could be identified. However, this product disappeared by adapting the final cooling step of the PCR reaction, suggesting that it represented a conformational variant (see **Supplementary Figure S1b**). The distribution of clones was: product 4 (canonical): 75 clones; product 1 and 1′: 8 clones; product 3: 6 clones; and product 2: 4 clones.

Next, exon internal reverse-transcriptase-quantitative PCR (RT-qPCR) analysis of exons 2 to 20 was performed to quantify aberrant splicing in cells from patient 1. All exons were expressed at similarly low levels of up to 12% compared with a healthy control (**Figure 1d**). The IVS1 *GAA* variant is known to allow 10–15% leaky wild type splicing.^9,15,30,31,32^ This suggests that the majority of *GAA* expression is derived from the IVS1 allele, and that the second allele containing the unknown *GAA* variant was expressed at much lower levels. Taken together, splicing analysis of all exons explained why patient 1 was diagnosed with Pompe disease, despite the lack of identification of the second *GAA* allele.

### The newly identified deep intronic variant c.2190-345A>G causes aberrant splicing in patient 1

To identify which *GAA* DNA variant could be responsible for aberrant splicing of exon 15 and 16, we first analyzed the sequences of the aberrantly spliced mRNA products 1–4. This failed to show any potentially pathogenic *GAA* variant. Then, we analyzed the genomic DNA surrounding the cryptic splices utilized in products 1–3. This revealed the presence of a heterozygous A > G variant at −1 relative to the cryptic splice acceptor site at c.2190–344 (**Figure 1e**). The intronic variant was missed by standard diagnostic sequence analysis because of its deep intronic location (**Figure 1f**, regions sequenced by standard diagnostics are indicated in gray). Its location close to the cryptic splice acceptor site suggested that it was involved in mediating the aberrant splicing observed in cells from patient 1.

**Figure 2:**
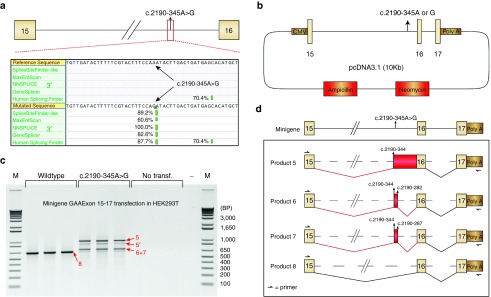
**Assessment of the pathogenic nature of the c.2190-345A>G variant.** (**a**) *In silico* prediction of the effect of the c.2190-345A>G variant on the generation of a 3′ cryptic splice site. Percentages indicate relative scores of strength per algorithm. (**b**) Cartoon of the minigenes containing the c.2190-345A or c.2190-345G variants. (**c**) Reverse-transcriptase-polymerase chain reaction (RT-PCR) analysis following transfection of minigenes into HEK293T cells. The reverse primer is specific for minigene mRNA as indicated in **d**. Products 5–8 indicate sequenced PCR products. Product 5′ refers to a secondary structural variant of product 5. Three biological replicates are shown. (**d**) Cartoon indicating the splicing products from **c** as identified by sequence analysis.

To test the effect of the c.2190-345A>G *GAA* variant on splicing, we used splicing prediction programs and minigenes. Alamut software, which includes five different algorithms to identify either 3′ or 5′ splice sites, predicted the generation of a strong splice acceptor site at c.2190–344 in the presence of the c.2190-345A>G variant (**Figure 2a**). This acceptor site was identical to the site identified in the aberrantly spliced products 1–3 of patient 1. Its predicted strength was similar to the strength of the canonical exon 16 splice acceptor site (compare **Figure 2a** and **Supplementary Figure S2a**). The region around the cryptic donor sites identified in products 1–3 was also scrutinized and this resulted in a moderately strong prediction of a splice donor site at c.2190–300 (used in product 3); a strong prediction of a splice donor at c.2190–287 (not detected *in vivo*); and a weak prediction (below the diagnostic threshold) of a splice donor site at c.2190–282 (used in product 2) (see **Supplementary Figure S2b**). Some of these potential splice donor sites were indeed utilized *in vivo* in a subset of mRNAs. In summary, splicing prediction programs uniformly predicted the generation of the new splice acceptor at c.2190–344 in response to the c.2190-345A>G variant, while prediction of splice donor sites was variable.

Next, minigenes were used to test the pathogenic nature of the c.2190-345A>G variant. Two minigenes including the genomic DNA region spanning *GAA* exon 15 to 17 were generated, one wild type (c.2190-345A) and one containing the variant (c.2190-345A>G, **Figure 2b**). These constructs were transfected in HEK293T cells, and *GAA* exon 16 splicing was analyzed using flanking exon RT-PCR. Endogenous HEK293T transcripts were circumvented by usage of a reverse primer specific to the minigene backbone. In the wild type minigene, a single product for amplification of exon 16 was detected that represented canonical exon 16 splicing (product 8, **Figure 2c**,**d** and **Supplementary Figure S2c**). This product was absent from untransfected cells, demonstrating that it was derived from the minigene. In contrast, the minigene containing the c.2190-345A>G variant failed to show expression of the canonical exon 16 splicing product, but showed expression of multiple alternative products (**Figure 2c**). The exon 16 PCR product was Topo cloned, and sequence analysis of 16 clones confirmed absence of the wild type product. Similar to endogenous exon 16 splicing in primary fibroblasts from patient 1, all aberrant products utilized the c.2190–344 splice acceptor in combination with various cryptic splice donor sites (**Figure 2d** and **Supplementary Figure S2c**). Product 5 contained intron retention from c.2190–344 toward exon 16 (three clones); product 6 showed usage of the cryptic splice donor at c.2190–282 (eight clones); product 7 used a cryptic splice donor at c.2190–287 (two clones) (**Figure 2d** and **Supplementary Figure S2c**). These findings indicate that the c.2190-345A>G variant causes aberrant splicing of *GAA* intron 15 and represents the new pathogenic *GAA* variant in patient 1. The unbiased analysis of splicing in cells from patient 1 has enabled the identification of aberrant splicing and the associated genomic DNA variant that was missed by standard diagnostic sequencing.

### Splicing correction by AONs in cells derived from patient 1 and a sibling, patient 2

**Figure 3:**
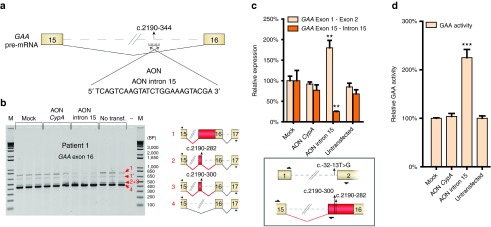
**Correction of aberrant splicing in fibroblasts from patient 1 using an antisense oligonucleotide (AON).** (**a**) Cartoon depicting the region in the *acid α-glucosidase* (*GAA*) pre-mRNA that was targeted with AON intron 15. (**b**) Flanking exon reverse-transcriptase-polymerase chain reaction (RT-PCR) analysis of *GAA* exon 16 in fibroblasts from patient 1 treated with AON intron 15. AON *CypA* was used as a control for AON treatment (see **Supplementary Figure S3**). Cartoons depict spliced mRNAs. Primer locations are indicated. Three biological replicates are shown. (**c**) RT-quantitative PCR (qPCR) analysis of the experiment outlined in **b**. Primers used for specific amplification of canonical (exon 1–exon 2) or aberrant (exon 15–intron 15) *GAA* mRNA are indicated in the cartoon below the graph. Data are normalized for *β-actin* and for mock transfection. Data represent mean ± SD of three biological replicates (**P = 0.01). (**d**) GAA enzymatic activity of patient 1 fibroblasts transfected with AONs. Data are normalized for mock transfection and represent the mean ± SD of three biological replicates (***P = 0.001).

Previously, AONs have been used to modulate pre-mRNA splicing. A relatively straightforward approach has been to block canonical splice sites to circumvent a variant hotspot and restore the reading frame, such as in DMD. AONs that bind to a splice site at the pre-mRNA can promote skipping of the splice site, which often results in utilization of another available (cryptic) splice site. We reasoned that a similar approach may be used to prevent utilization of a cryptic splice site such as the one generated by the c.2190-345G>A variant in patient 1. To test this, we first analyzed the feasibility of AON-mediated splicing modulation of pre-mRNA in primary fibroblasts. A control phosphorodiamidate morpholino oligonucleotide (PMO)-based AON was designed that targets the splice donor site of exon 4 in *Cyclophilin A* (*CypA*) pre-mRNA (AON *CypA*) (see **Supplementary Figure S3a**; based on ref. 33). AON *CypA* efficiently promoted skipping of *CypA* exons 3 and 4 in fibroblasts from patient 1 as analyzed by RT-PCR (see **Supplementary Figure S3b**) and RT-qPCR (see **Supplementary Figure S3c**). This demonstrated robust splicing modulation using PMO-based AONs in primary fibroblasts. Next, we designed an AON that targeted the cryptic splice site at c.2190–344 (AON intron 15; **Figure 3a**). Transfection of AON intron 15 in fibroblasts from patient 1 resulted in lower levels of endogenous aberrant splice products 1, 2, and 3, and higher abundance of canonical splice product 4, as shown by semiquantitative exon flanking RT-PCR analysis (**Figure 3b**). Mock transfection or transfection with AON *CypA* showed no effect. Quantitative RT-qPCR analysis was then performed to quantify changes in *GAA* splicing. Changes in wild type splicing were quantified using primers spanning the *GAA* exon 1-exon 2 splice junction. Low-abundant aberrantly spliced products were quantified using a forward primer annealing to exon 15 and a reverse primer that anneals to the common intronic region included in all aberrant splice products 1–3 (**Figure 3c**, lower panel). Treatment with AON intron 15 promoted splicing toward canonical *GAA* mRNA, while it inhibited aberrant *GAA* splicing (**Figure 3c**). Mock transfected cells or cells transfected with AON *CypA* showed no effect on *GAA* splicing. The effect of AON intron 15 treatment on GAA enzymatic activity was then assessed, and this showed a more than twofold enhancement (**Figure 3d**). Importantly, this increase would result in a residual enzymatic activity that is above the disease threshold of 20% of control, suggesting that the AON intron 15-mediated splicing correction was sufficient to alleviate Pompe disease in cells from patient 1.

A sibling of patient 1, patient 2, was also diagnosed with Pompe disease. This patient contained the IVS1 *GAA* variant but also lacked identification of the second pathogenic *GAA* allele using standard diagnostic analysis. Genomic DNA analysis showed that the unknown allele of patient 2 was c.2190-345A>G, identical to the second allele of patient 1 (see **Supplementary Figure S4a**). Flanking exon RT-PCR analysis of exon 16 in primary fibroblasts indicated the same aberrant splicing products compared with patient 1 (see **Supplementary Figure S4b**). Treatment with AON intron 15 resulted in a similar correction of splicing compared with patient 1, as shown by RT-PCR (see **Supplementary Figure S4b**) and RT-qPCR (see **Supplementary Figure S4c**). Also the GAA enzymatic activity was enhanced by AON intron 15 treatment to 250% compared with mock transfection (see **Supplementary Figure S4d**), similar to patient 1. The results with patient 2 confirm the feasibility of splicing correction by repression of the newly formed, deep intronic cryptic splice site at c.2190–344.

### Inhibition of a natural intronic cryptic splice acceptor to correct splicing in cells derived from patient 3

To test whether the blocking of cryptic splice sites by AONs may represent a general approach to correct splicing in Pompe disease, we examined two additional patients.

**Figure 4:**
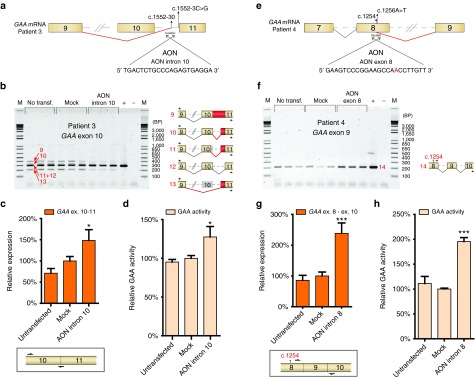
**Antisense oligonucleotides (AON)-mediated correction of cryptic splicing in fibroblasts from two additional patients.** (**a**) Cartoon depicting the region in the *acid* *α-glucosidase* (*GAA*) pre-mRNA of patient 3 that was targeted with an AON. This patient utilizes a cryptic splice site at c.1552-30 due to a homozygous variant c.1552-3C>G as described previously.^15,35^ The sequence of AON intron 10 is shown. (**b**) Flanking exon reverse-transcriptase-polymerase chain reaction (RT-PCR) analysis of *GAA* exon 10 in fibroblasts from patient 3 that were treated with AON intron 10. Cartoons depict spliced mRNAs. Primer locations are indicated. Three biological replicates are shown. + represents analysis of a healthy control. (**c**) RT-quantitative PCR (RT-qPCR) analysis of *GAA* exon 10–exon 11 expression in fibroblasts from patient 3 treated with AON intron 10. The forward primer anneals to *GAA* exon 10 and the reverse primer anneals to the *GAA* exon 10–11 junction for specific detection of canonical exon 10–11 splicing. Data are normalized for *β-actin* and for mock transfection and represent mean ± SD of three biological replicates (*P = 0.05). (**d**) GAA enzymatic activity in patient 3 fibroblasts transfected with AON intron 10. Data represent mean ± SD of three biological replicates (*P = 0.05). (**e**) Cartoon depicting the region in the *GAA* pre-mRNA of patient 4 that was targeted with an AON. This patient utilizes a cryptic splice site at c.1254 due to the c.1256A>T variant as described previously.^15^ The sequence of AON exon 8 is shown. **(f)** Flanking exon RT-PCR analysis of *GAA* exon 9 on fibroblasts from patient 4 transfected with AON exon 8. Primers indicated in the cartoon specifically amplify canonically spliced *GAA* exon 8 mRNA from the allele harboring the c.1256A>T variant. Three biological replicates are shown. + represents analysis of a healthy control. **(g)** RT-qPCR analysis of *GAA* exon 8 – exon 10 expression in fibroblasts from patient 4 treated with AON exon 8. Primers only amplify canonically spliced *GAA* exon 8 mRNA from the allele harboring the c.1256A>T variant. Data represent mean ± SD of three biological replicates (***P = 0.001). (**h**) GAA enzymatic activity in patient 4 fibroblasts transfected with AON exon 8. Data represent mean ± SD of three biological replicates (***P = 0.001). Reprinted from refs. 15 and 35 with permission of the publisher.

Patient 3 (**Table 1**) represents an example in which an intronic *GAA* variant (homozygous c.1552-3C>G,^15,34^) weakens a canonical splice site (splice acceptor of exon 11), which promotes utilization of a nearby cryptic splice site (c.1552-30, **Figure 4a**). We hypothesized that blockage of this cryptic splice site with an AON may enhance the probability of utilizing the canonical splice site. The details of aberrant splicing in fibroblasts of this patient have been described by us previously (patient 6 in ref. 15). We now performed Topo cloning of cDNA products derived from flanking exon PCR of exon 10 (see **Supplementary Table S1**, -AON), and this resulted in the identification of two additional splicing products (10 and 11 in **Figure 4b**). All identified splicing products include: retention of intron 10 (product 9; reading frame disrupted), splicing from the canonical splice donor site at exon 10 to a natural cryptic splice acceptor at c.1552-30 in intron 10 (product 10, reading frame intact), splicing from a natural splice acceptor at c.1537 in exon 10 to a cryptic splice acceptor at c.1552-30 (product 11; reading frame intact), leaky wild type splicing (product 12), and skipping of exon 10 plus utilization of the cryptic splice acceptor at c.1552-30 (product 13; reading frame intact) (**Figure 4b** cartoons and **Supplementary Figure S5a**).

AON intron 10 was designed to target the cryptic splice site at c.1552-30 (**Figure 4a**). Transfection of this AON in fibroblasts from patient 3 caused a reduction of expression of aberrantly spliced mRNAs (products 9–11 and 13), while canonical spliced mRNA (product 12) seemed enhanced, as analyzed by flanking exon PCR of exon 10 (**Figure 4b**). This was also evident from the numbers of Topo clones derived from this PCR: without AON treatment, 49% of clones contained wild type exon 10 spliced cDNA, while treatment with AON intron 10 yielded 80% wild type cDNA clones (see **Supplementary Table S1**). It should be noted that product 9 (full intron 10 retention) is out of frame and is likely subject to mRNA degradation, which prevents an estimation of the total amount of transcripts produced. Both flanking exon RT-PCR and Topo cloning can only be considered semiquantitative methods. To quantify the effect of AON intron 10, RT-qPCR analysis was performed using a primer that anneals to the boundary of exon 10/11, as this distinguishes wild type from aberrant products (**Figure 4c**, lower panel). This confirmed that AON intron 10 enhanced canonical exon 10 splicing with 50% (**Figure 4c**). In agreement, a modest but significant increase of 1.3-fold of GAA enzymatic activity was detected in cells treated with AON intron 10 (**Figure 4d**). This patient has a considerable residual GAA activity (12.6 nmol/hour/mg, representing ~10% of healthy control values), and an enhancement of 130% elevates GAA activity to levels closer to the disease threshold of ~20% of healthy control. Taken together, these results show that aberrant splicing caused by a splice site variant can be at least partially corrected by preventing the utilization of a nearby located cryptic splice site using AONs.

### Correction of aberrant splicing from a newly formed exonic splice donor site in cells derived from patient 4

To test whether it is also possible to modulate splicing by blocking a newly formed cryptic splice site formed at an exonic location, we examined patient 4. This patient carries the c.1256A>T missense *GAA* variant on allele 1, which leads to the generation of an exonic cryptic splice donor site at c.1254 in exon 8 (patient 8 in ref. 15; **Figure 4e**, **Supplementary Figure S5b**). Products from this allele include skipping of the 3′ part of exon 8. The second allele of this patient contains another *GAA* splicing variant, c. 1551+1G>T, which causes skipping of exon 10 (ref. 15; **Supplementary Figure S5b**). We hypothesized that blocking of the cryptic splice site at c.1254 with an AON may restore normal splicing of the c. 1256A>T allele. First, we tested the possibility that the c.1256A>T missense (p.D419V) variant, which would still be present in the splice-corrected mRNA, affects GAA enzymatic activity. To test this, the *GAA* cDNA was cloned into an expression vector, and the c.1256A>T variant was introduced by site directed mutagenesis. Transfection into HEK293T cells followed by measurement of GAA enzymatic activity showed that the c.1256A>T variant did not impair GAA enzymatic activity (see **Supplementary Figure S5c**). This suggests that its pathogenic effect can be solely attributed to its effects on pre-mRNA splicing. Next, AON exon 8 was designed complementary to the cryptic splice site including the variant at c.1256 (**Figure 4e**, variant sequence in red). AON exon 8 was transfected in primary fibroblasts from patient 4, and the effect on *GAA* mRNA expression and GAA enzymatic activity was determined. Flanking exon PCR was performed for exon 9 using primers designed to specifically detect splicing correction from the c.1256A>T allele with minimal interference from the c.1551+1G>T allele. This was achieved by using a forward primer that anneals to the 3′ part of exon 8, which is largely skipped in mRNA from the c.1256A>T allele, and a reverse primer that anneals to exon 10, which is completely skipped in mRNA from the c.1551+1G>T allele (**Figure 4f**). AON exon 8 enhanced the expression of canonically spliced exon 8 from the c.1256A>T allele (product 14, **Figure 4f**). Quantitative analysis using RT-qPCR with similar allele-specific primers confirmed that AON exon 8 enhanced expression of the c.1256A>T allele with 2.3-fold (**Figure 4g**). Besides promoting canonical exon 8 splicing, AON exon 8 also caused skipping of exon 8, as shown by flanking exon PCR of exon 8 (see **Supplementary Figure S5d**). The net result of this effect was a partial correction of the splicing effect of the c.1256A>T variant. This is further discussed in the legend to **Supplementary Figure S5d**.

In agreement with the mRNA analysis, GAA enzymatic activity was increased 2 fold as the result of AON exon 8 treatment (**Figure 4h**). This patient showed a relatively low GAA enzymatic activity of 5.4 nmol/hour/mg (4.4% of healthy control) and a juvenile disease onset. Elevation of the GAA enzymatic activity with a factor 2 is expected to attenuate the onset and severity of Pompe disease. In summary, it is possible to redirect aberrant splicing toward canonical splicing by targeting a cryptic splice site formed by a variant at an exonic location.

## Discussion

### Assay for splicing analysis and genotyping

**Figure 5:**
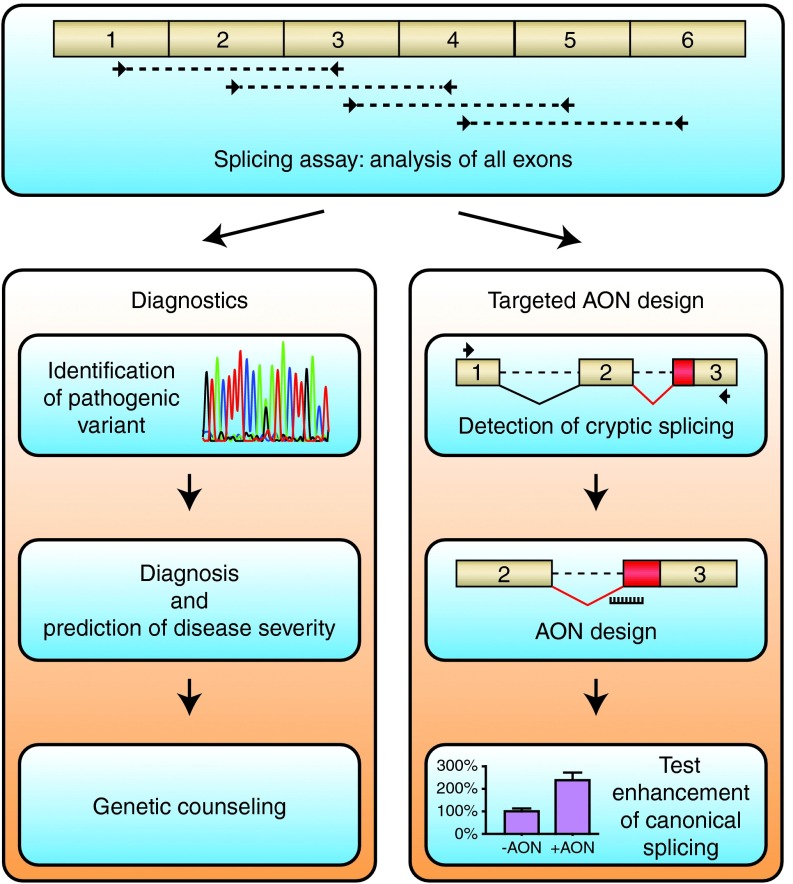
**Pipeline for the identification and targeting of aberrant splicing events as applied to Pompe disease.** The splicing assay consists of unbiased mRNA analysis of all coding exons by reverse-transcriptase-quantitative PCR and Sanger sequencing.^15^ The results aid in diagnostics (left panel), but also provide the basis for the development of antisense oligonucleotides (AONs) that repress the utilization of natural or newly formed cryptic splice sites (right panel). Reprinted from ref. 15 with permission of the publisher.

The assessment of a gene of interest for possible splicing defects can provide both qualitative and quantitative information on aberrant splicing.^15^ This is important for establishing the pathogenic nature of a variant, with implications for diagnostics, genetic counseling, prediction of disease severity, and the development of novel treatment options. The splicing assay can also be used to identify deep intronic variants that have been missed by standard diagnostic DNA sequencing. Especially in countries in which a diagnosis requires the identification of two pathogenic variants to be eligible for therapy, as is the case with enzyme replacement therapy for Pompe disease, this information can have a serious impact on treatment options for the patient. A rather straightforward flow has been applied here to identify a deep intronic variant. This started with the detection of aberrant splicing using unbiased, PCR-based analysis of all exons (**Figure 5**). It is important to note that also small amounts of aberrant mRNA products should be analyzed as these may be out of frame and subject to mRNA decay, as is the case in patient 1. Next, the region surrounding the novel splice sites was sequenced at the genomic DNA level. This revealed a potential splicing variant. Third, the pathogenic effect of the variant was confirmed by *in silico* analysis and using a minigene construct (**Figure 5**). This approach improves standard diagnostic practice and provides a potential basis for therapeutic development.

The use of RNA sequencing-based methods for splicing analysis of patients may play an increasingly important role in the future.^35,36,37^ However, at present, there are several disadvantages of using RNA sequencing-based techniques for diagnostics. In particular, to fully assess aberrant splicing of a particular gene in a quantitative manner, deep sequencing combined with bioinformatics analysis is required, which is rather expensive. The present PCR-based assay can be standardized and is fast, cheap, and sensitive. A useful addition may be the implementation of new techniques such as single molecule real-time DNA sequencing.^38^ This could be applied to cDNA, for example to determine whether pathogenic variants reside on the same allele or are compound heterozygous, a question that could not be addressed for patients 1 and 2 due to the absence of DNA from the parents.

### Disturbance of the splicing equilibrium by point variants

It is intriguing that a single point variant can generate a strong splice site as is the case for patient 1. This is in line with the fact that the sequence requirements for the generation of a potential splice site are relatively limited.^13^ The decision to utilize a splice site can depend on many aspects of gene expression, including the speed of RNA pol II transcription, cis-acting sequences in the pre-mRNA, pre-mRNA structure, chromatin modifications, and expression levels of splicing proteins.^13,14^ It is therefore very difficult to predict the outcome of splicing based on *in silico* analysis of splice site strength. In patient 1 and 2 the formation of a new 3′ splice site not only results in retention of the downstream intronic sequence, but also in the utilization of two natural cryptic 5′ splice sites (see **Supplementary Figure S2b**). The 10–15% leaky wild type splicing detected in these patients is likely derived from the IVS1 allele, which is known to have this level of normal splicing.^9,15,30,31^ It should be noted that we could not formally prove that the IVS1 and c.2190-345A>G were compound heterozygous variants, as genomic DNA from the parents of patients 1 and 2 could not be obtained. However, the combined evidence obtained here strongly suggests that this is the case. In patient 3, the weakening of the canonical 3′ splice site of exon 11 is straightforward, but the outcome with at least 3 different splicing products and the involvement of a nearby cryptic splice site was difficult to anticipate.^15^ Patient 4 illustrates that missense variants can also affect splicing. In fact, recent evidence suggests that this phenomenon is rather frequent and has been estimated to be the case in ~20% of all missense variants.^2,3,4^ In this patient, aberrant splicing appeared to be solely responsible for the pathogenic effect of the c.1256A>T variant, as the enzymatic activity of the mutated protein was similar to wild type *GAA* in cDNA expression analysis. Taken together, point variants can induce complex changes in alternative splicing that are difficult to predict but can be characterized using PCR-based analysis of all exons.

### Targeted splicing correction using AONs

The information obtained with the splicing assay can directly be used to design AONs directed toward inhibition of cryptic splicing by targeting the identified cryptic splice site (**Figure 5**). This was done in the case of patient 1 and 2. In primary fibroblasts from these patients, the enhancement of GAA enzymatic activity after treatment with AON was sufficient to reach levels above the Pompe disease threshold of 20% activity of healthy controls. This is further illustrated by the experience in our diagnostic center, at which all Pompe patients in the Netherlands are diagnosed. A combination of clinical and biochemical parameters applied to >200 patients and >300 healthy individuals showed a patient range to be 0–20 nmol/hour/mg, and a normal range from 40–180 nmol/hour/mg (ref. 39 and diagnostics department, Erasmus MC). This suggests that the AON designed to correct splicing in both patients has the potential to restore GAA enzyme levels toward healthy control levels. It should be noted that the correction of glycogen storage by AONs in cells in culture cannot be studied at present, because cells from juvenile/adult onset Pompe patients do not show glycogen accumulation *in vitro*. This may imply that additional factors are required that are involved in lysosomal pathology in juvenile/adult onset Pompe disease. In agreement, several studies including our own report on the strongly heterogeneous disease onset and progression of juvenile/adult Pompe patients, also when they have identical *GAA* genotypes.^40,41,42^

A priori, it was not always obvious that AON mediated repression of cryptic splicing would enhance canonical splicing. In the case of patients 1 and 2, we anticipated that repression of a deep intronic, newly formed splice site would restore canonical splicing, as was indeed observed. However, patients 3 and 4 represented more challenging cases.

In patient 3, the point variant was located near a 3′ splice site, which resulted in weakening of the canonical splice site and utilization of the c.1552-30 cryptic splice site. Another observed event as a consequence of the c.1552-3C>G variant was retention of intron 10. It was not clear whether blocking of the cryptic splice site with an AON would promote canonical splicing or intron retention. This depended on the question whether the canonical splice site was still partially functional despite the nearby c.1552-3C>G variant. In this case, the AON blocked utilization of the cryptic splice site without promoting other detectable forms of aberrant splicing. This showed that the canonical splice site was still partially functional, suggesting a competition model in which the strongest nearby splice site is preferred. Competition for splice site usage is thought to play an important role in the outcome of pre-mRNA splicing, as is the distance to the next available splice site.^13,14^ The fact that repression of the natural upstream cryptic splice site promoted splicing to a weakened canonical splice site is encouraging and may suggest a general strategy for similar cases.

In patient 4, the targeting of an AON to an exonic region represented a challenge, as this has the potential to interfere with protein translation.^43^ We found no evidence for this, as canonical splicing was increased with a concomitant increase in GAA protein activity. However, the AON targeted to exon 8 resulted in a new equilibrium of AS, in which both canonical splicing and aberrant splicing were altered. This illustrates that AON targeting of cryptic splice sites may induce new splice forms, and it underscores the importance of testing for this possibility. In this patient, the AON-induced changes in AS were advantageous as the c.1256A>T allele showed an improvement to 195%, while the effect on exon 8 splicing of the second allele was irrelevant as this already was affected by aberrant splicing of exon 10.

### Application of AONs in human disease

To translate the present results to a clinical setting, more preclinical testing is required. Extensive work has been performed for a number of disorders including spinal muscular atrophy and DMD (at the level of clinical trials), and Hutchinson-Gilford progeria syndrome^19^ and type I Usher syndrome^20^ (using animal models). These studies have shown that AONs can enhance expression of the gene of interest following various routes of administration. It is important to consider the disease-specific aspects for AON-based treatment options. For instance, in the case of DMD, the maximal effect that can be reached using an exon skipping strategy is the milder Becker's Muscular Dystrophy phenotype due to the expression of a truncated dystrophin protein, and this is still a serious condition. In addition, in DMD or Becker's Muscular Dystrophy, not only skeletal muscle is affected, but also cardiac muscle and the central nervous system, which are more difficult to target using AONs. Cellular uptake can be further enhanced by conjugation with cell penetrating peptides or octaguanidine dendrimers, which have been applied to PMO-based AONs and showed good efficacy in animal models.^44,45,46^ In contrast to the situation in DMD, AONs in Pompe disease have the potential to restore wild type *GAA* expression toward levels present in healthy individuals. In the childhood/adult form of the disease, skeletal muscle cells are the major cells affected without involvement of cardiac or neuronal cells.^47,48^ This contrasts with classic infantile Pompe disease, in which hypertrophic cardiomyopathy is present at birth,^25^ and cognitive decline may progressively develop.^49,50^ This study identifies AONs for childhood/adult onset Pompe patients. Further studies are required to test the efficacy of these AONs to target skeletal muscle cells *in vitro* and *in vivo*. Interestingly, a recent study used AONs with a PMO backbone coupled to an arginine-rich cell penetrating peptide to target muscle glycogen synthase in a mouse model for Pompe disease, suggesting that it is feasible to use AONs for Pompe disease *in vivo*.^46^ The ongoing development of methods to enhance tissue delivery of AONs *in vivo* by changing the chemistry,^51^ charge,^52^ by coupling to cell penetrating peptides,^21,22,23,44–46^ or by using carriers such as exosomes or nanoparticles,^53,54^ is expected to further stimulate clinical testing of AONs. Current knowledge from recent clinical trials on the safety of AON backbones should help to facilitate further development of AONs, and to test whether AONs provides a valuable alternative or addition to enzyme replacement therapy in Pompe disease.

## Materials and methods

*Materials.* Fetal Bovine Serum was purchased from GE Healthcare (Logan, UT). Penicillin/Streptomycin/Glutamine (p/s/g) and TryplE were purchased from Thermo Fisher Scientific (Waltham, MA). DMEM High Glucose was purchased from Lonza (Walkersville, MD). DMSO Endoporter reagent and PMO-based AONs were purchased from Gene-Tools (Philomath, OR) and all other chemicals were purchased from Sigma Aldrich (Irvine, UK) unless otherwise stated.

*Patients.* Patients were diagnosed with Pompe disease at the Center for Lysosomal and Metabolic Diseases of the Erasmus MC, Rotterdam, The Netherlands. Diagnosis was based on GAA enzymatic activity in leukocytes and/or fibroblasts, *GAA* variants, and clinical symptoms. Analysis was performed on anonymous patient material using informed consent.

*Nomenclature.* All references toward locations of variants and splice sites are made according to HGVS standards (http://www.hgvs.org/mutnomen/).^55^ The reference GAA transcript was RefSeq NM_000152.3.

*Generation of minigene and cDNA construct.* For generation of the minigene containing the genomic *GAA* DNA region of *GAA* exon 15–17 (chr17:80113219-80117749, GRCh38/hg38), genomic DNA from a healthy control was amplified with PFU Ultra Hotstart polymerase (Agilent Technologies, Santa Clara, CA) and cloned in the pcDNA3.1(−)Myc-His A vector using the XbaI and NotI restriction sites. The c.2190-345A>G variant was introduced using the QuikChange II Site-Directed Mutagenesis Kit (Agilent Technologies). The *GAA* cDNA expression construct (RefSeq NM_000152.3) was generated in the same vector, using restriction sites NheI and AflII, and the c.1256A>T variant was introduced using site directed mutagenesis as above. All constructs were verified by sequence analysis (all primers used in **Supplementary Table S2**).

*Cell culture and transfections.* HEK293T cells and primary human fibroblasts were cultured in 10% FBS, 1× p/s/g and DMEM High Glucose. Transfection of minigenes and cDNA constructs was performed using Lipofectamine 2000 according to the manufactures' protocol. Cells were harvested 48 hours after transfection. *GAA* cDNA expression was corrected for mRNA expression of the *Neomycin* cassette present on the pcDNA3.1 backbone using RT-qPCR analysis. Transfection of AONs was performed using 4.5 µl/ml endoporter in the medium at a concentration of 20 µmol/l AON. RNA and protein were harvested 3 and 5 days after transfection, respectively.

*Splicing assay.* The splicing assay was performed as described before.^15^ In short, RNA was isolated using the RNAeasy miniprep kit (Qiagen, Germantown, MD). RT-PCR was performed with 800 ng RNA input using iScript (Biorad, Hercules, CA) and FastStart Taq Polymerase (Roche, Penzberg, Germany). qPCR was carried out using iTaq SYBR green supermix (Biorad,), and was performed on a cfx96rts cycler (Biorad). Primers are shown in **Supplementary Table S2**. *β-actin* was used as a reference gene. All primer sets used showed high efficiency and specificity based on melting-curve analysis and standard curve measurements.

**In silico* splice prediction.* *In silico* prediction was carried out using Alamut Visual version 2.6.1, which uses five algorithms for predicting 5′ and 3′ splice junctions (description of algorithms at http://www.interactive-biosoftware.com/doc/alamut-visual/2.6/splicing.html) as described.^15^

*GAA enzymatic activity.* Enzymatic activity of the GAA protein was determined as previously described.^33^ In short, cell lysates were incubated with 4-methyl-umbelliferyl-α-d-glucopyranoside (4-MU, Sigma) in citrate–phosphate buffer for 1 hour at 37°C, after which fluorescence was measured at 365/448 nm with the Varioskan system (Thermo Fisher). The GAA enzymatic activity is dependent on cell culture conditions. To correct for this, a control cell line was included in each experiment, and the GAA enzymatic activity was normalized based on this control line.

**SUPPLEMENTARY MATERIAL** **Figure S1.** Sequence analysis and structural effects of *GAA *splicing products 1–4 identified in cells from patient 1. **Figure S2.** *In silico* prediction and sequence analysis of *GAA *splicing products 5–8 in cells from patient 1. **Figure S3.** Exon skipping in *CypA* pre-mRNA by an AON. **Figure S4.** AON intron 15 restores *GAA* splicing in cells from patient 2, a sibling of patient 1. **Figure S5.** Further analysis of patients 3 and 4. **Table S1.** Number of products with and without AON treatment of patient 1. **Table S2.** Primers used for experiments.

## Figures and Tables

**Table 1:**
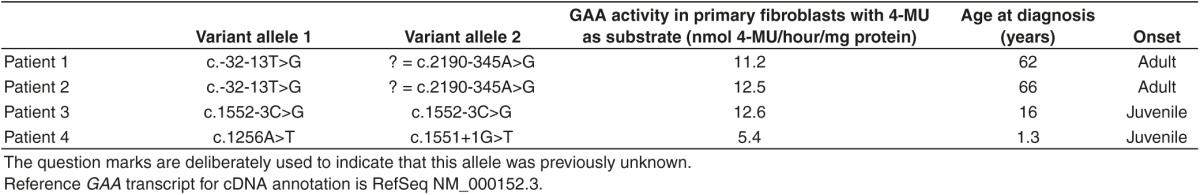
Patient characteristics
