# Supplementary material for: From Cryptic Toward Canonical Pre-mRNA Splicing in Pompe Disease: a Pipeline for the Development of Antisense Oligonucleotides
Source: Mol Ther Nucleic Acids. 2016 Sep 13;5(9):e361–. doi: 10.1038/mtna.2016.75 (PMC5056997; doi:10.1038/mtna.2016.75)
Supplement: Supplementary Figures and Tables [file mtna201675x1.pdf]

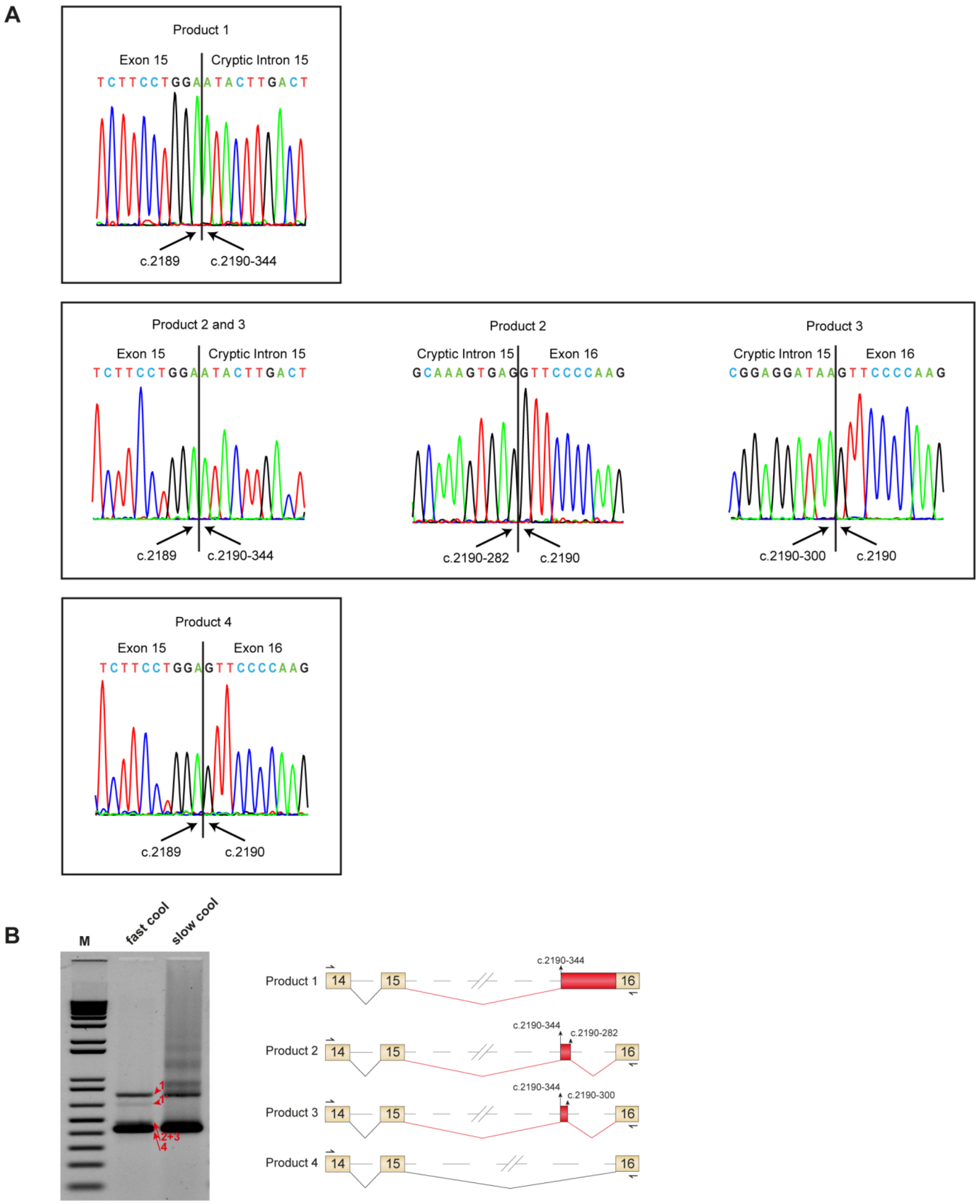

Supplementary Figure 1. Sequence analysis and structural effects of *GAA* splicing products 1-4 identified in cells from patient 1. **(A)** Sequence analysis of splice junctions utilized in splice products 1-4, shown in Fig 1C. **(B)** Effect of the speed of cooling on formation of secondary structure in PCR products. A sample from the flanking exon RT-PCR analysis of *GAA* exon 16 on primary fibroblasts from patient 1 is shown. Left lane: fast cooling, right lane: slow cooling. Product 1' disappeared as a consequence of slow cooling, suggesting that it represents a conformational variant. Cartoons of products 1-4 are shown in the right panel.

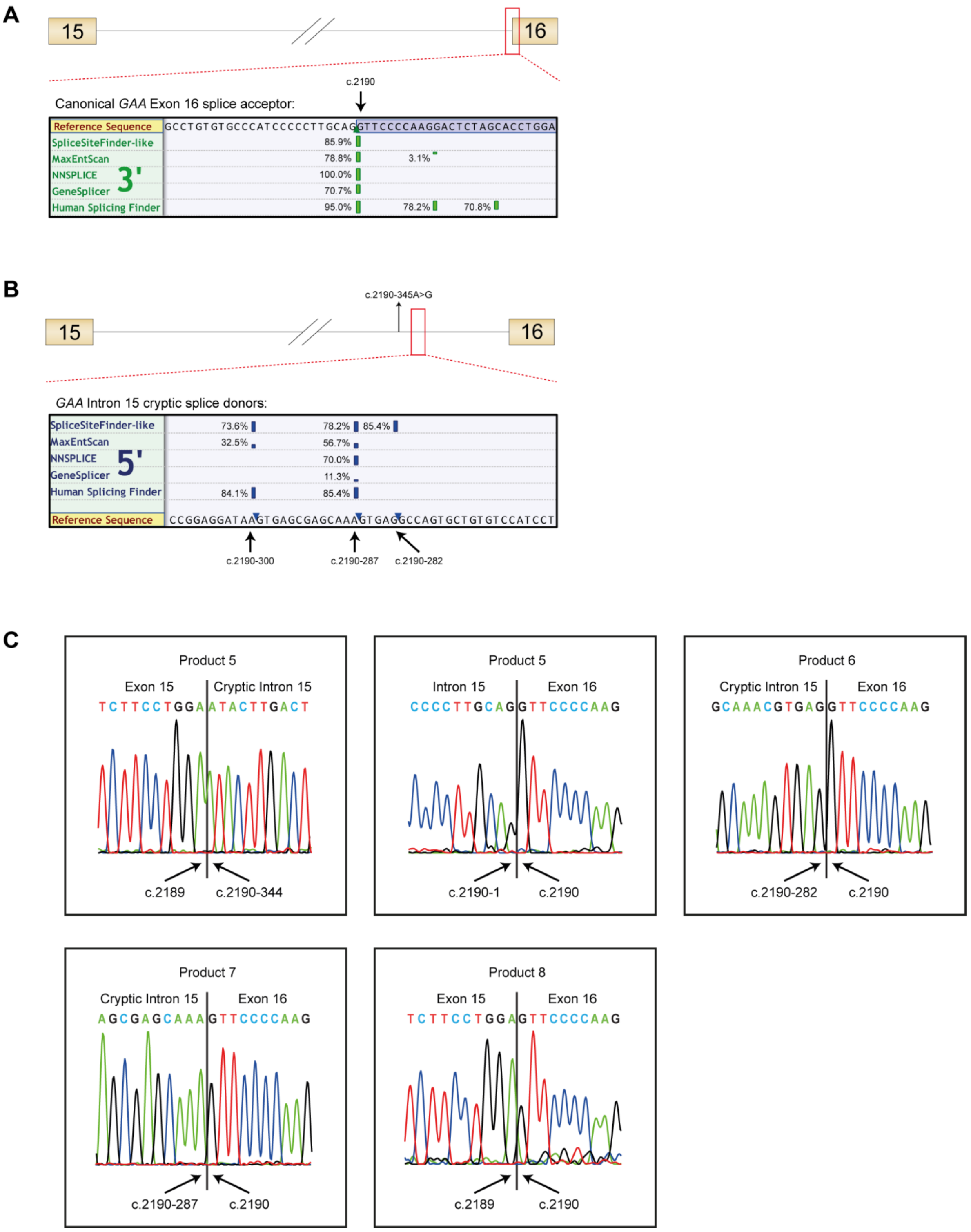

Supplementary Figure 2. In silico prediction and sequence analysis of *GAA* splicing products 5-8 in cells from patient 1. **(A)** In silico prediction of the 3' canonical splice acceptor site located at the *GAA* intron 15 – exon 16 junction. **(B)** In silico prediction of the 5' cryptic splice donor sites located in *GAA* intron 15 that were found to be utilized in the context of the c.2190-345A>G variant. **(C)** Sequence analysis of splice junctions utilized in products 5-8, shown in Fig 2D.

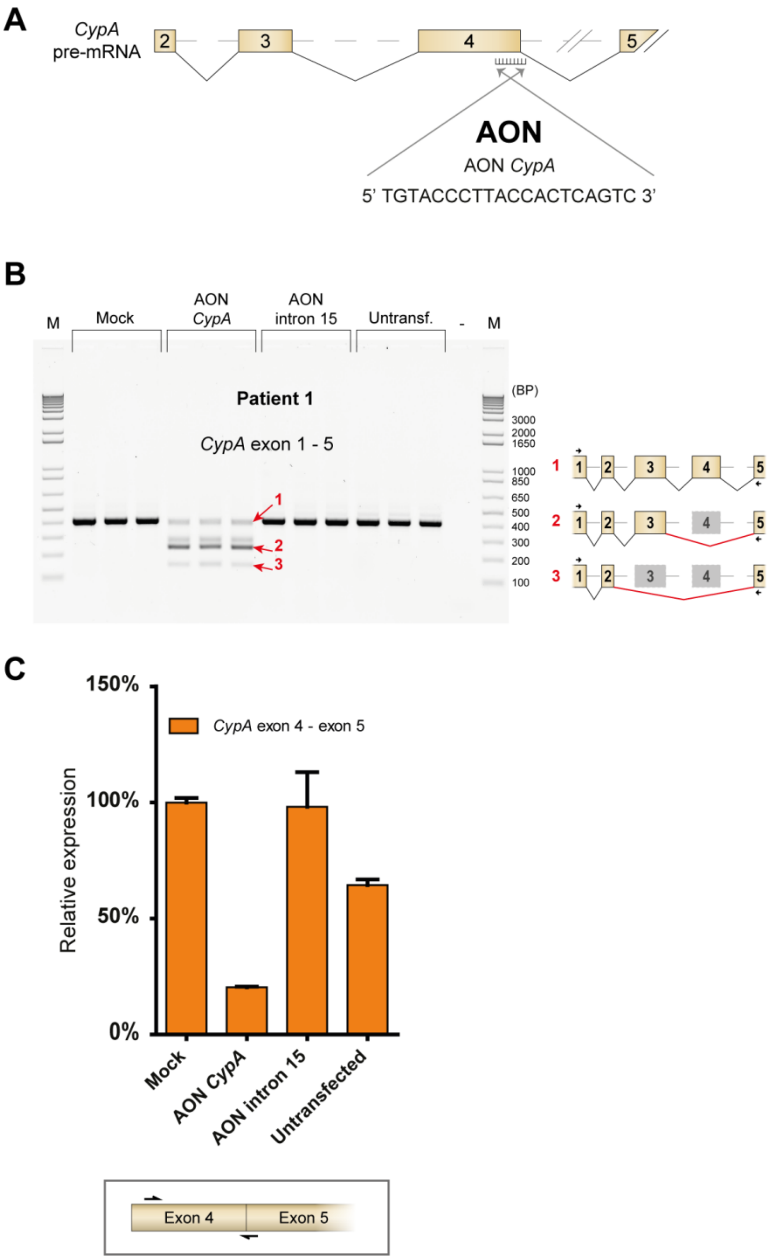

Supplementary Figure 3. Exon skipping in *CypA* pre-mRNA by an AON. **(A)** Cartoon depicting the region in the *CypA* pre-mRNA that was targeted with an AON. The sequence of AON *CypA* is shown. **(B)** Flanking exon RT-PCR analysis of fibroblasts from patient 1 treated with AONs. Cartoons depict spliced mRNAs. Primer locations are indicated. Three biological replicates are shown. **(C)** RT-qPCR analysis of the experiment in **(B)**, using primers spanning the *CypA* exon 4-5 splice junction (shown in the lower panel). Data are normalized for *ß-Actin* and for mock transfection and represent means +/- SD of three biological replicates.

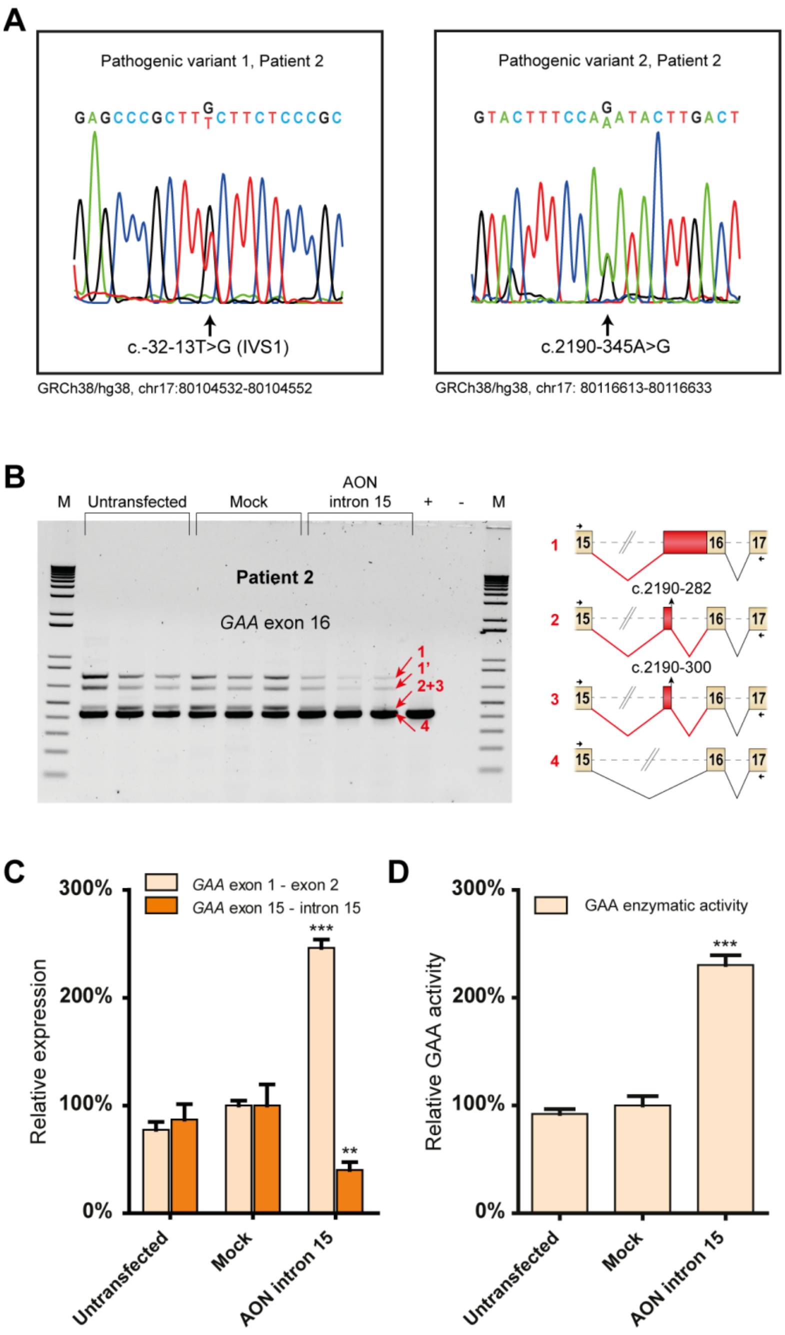

Supplementary Figure 4. AON intron 15 restores *GAA* splicing in cells from patient 2, a sibling of patient 1. **(A)** Sequence analysis of genomic DNA obtained from primary fibroblasts of patient 2. **(B)** Flanking exon RT-PCR analysis of exon 16 in primary fibroblasts from patient 2 treated with AON intron 15. Products were inferred from the analysis of patient 1 based on electrophoretic mobility, and these are indicated in the cartoons along with primer locations in the right panel. Three biological replicates are shown. **(C)** RT-qPCR analysis of the experiment in **(B)**. **(D)** Effect of AON intron 15 on GAA enzymatic activity in fibroblasts from patient 2. Data in **(C)** and **(D)** represent means +/- SD of three biological replicates (** p = 0,01 , *** p = 0,001).

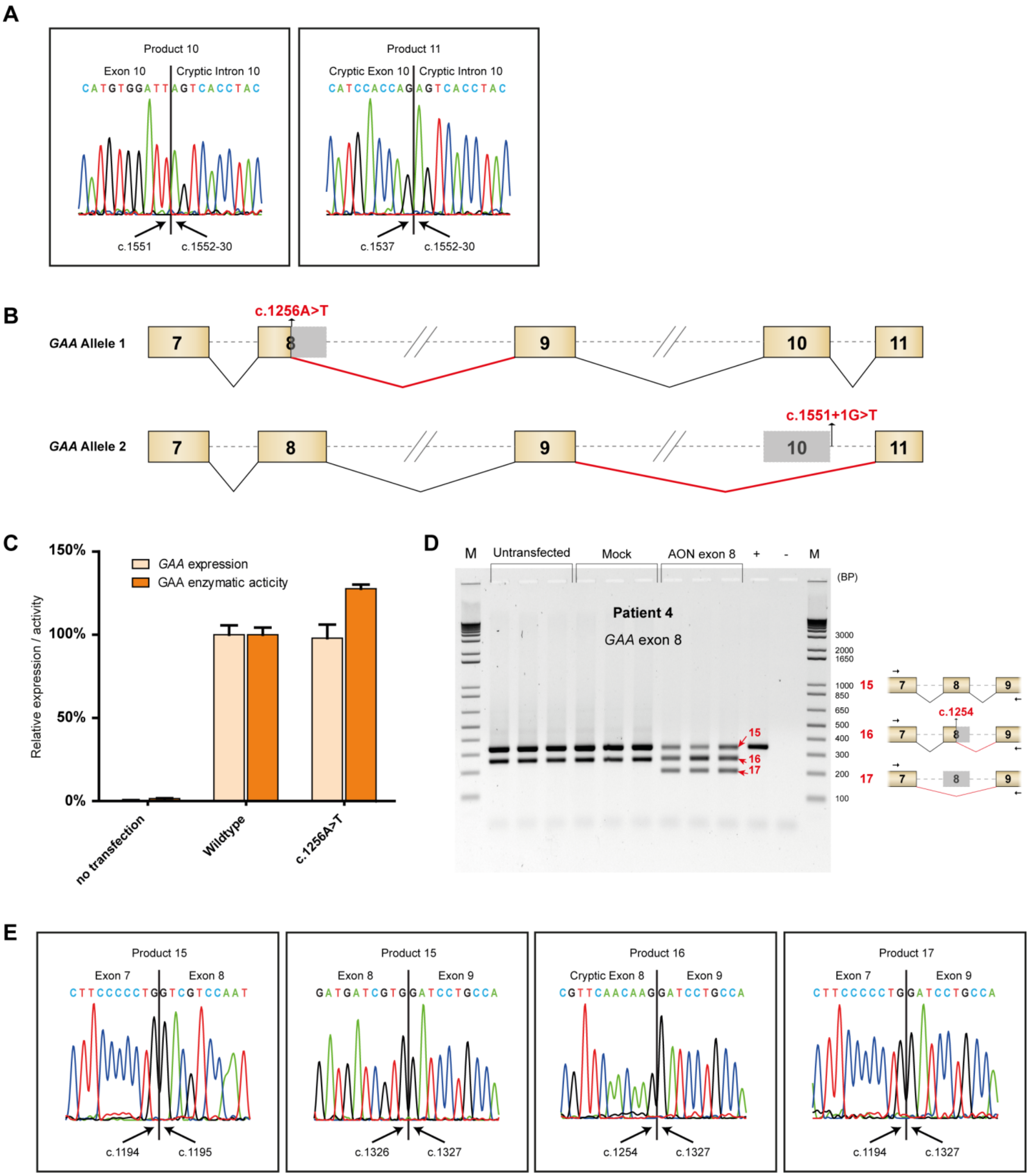

Supplementary Figure 5. Further analysis of patients 3 and 4. **(A)** Sequence analysis of newly identified products 10 and 11 derived from patient 3 (see Fig. 4B). **(B)** Cartoons of the *GAA* alleles of patient 4. **(C)** Effect of the c.1256A>T variant on *GAA* cDNA mRNA expression and enzymatic activity. Wild type or mutant cDNA constructs were transfected in HEK293T cells. GAA enzymatic activity was determined using 4-MU as substrate. *GAA* mRNA expression was also determined (using RT-qPCR analysis) to verify that both constructs were expressed at similar levels, which was the case. Neomycin mRNA expression (using RT-qPCR analysis) was used to normalized for transfection efficiency. Data are expressed relative to wild type *GAA* mRNA expression/activity. Both expression and protein activity data represent means +/- S.D. of three biological replicates. **(D)** Flanking exon RT-PCR of patient 4

GAA exon 8 after treatment. Cartoons depict spliced mRNAs. Primer locations are indicated. Three biological replicates are shown. + represents cDNA from a healthy control. Flanking exon RT-PCR analysis of *GAA* exon 8 showed that AON exon 8 had multiple effects on exon 8 splicing, resulting in similarly abundant expression of three products: mRNA with canonically spliced exon 8 (product 15), mRNA in which the 3' part of exon 8 was skipped via utilization of the cryptic splice site at c.1254 (product 16) and complete skipping of exon 8 (product 17) (for sequence see **(E)**). To explain why AON exon 8 corrected splicing from the c.1256A>T allele while at the same time it also caused aberrant splicing of total *GAA* expression, the effects of AON exon 8 on the individual alleles should be considered. Without AON treatment, the c.1256A>T allele shows predominant skipping of the 3' part of exon 8 (assume here 95%), and a small amount of leaky wild type splicing (assume here 5%). After AON exon 8 treatment, the splicing equilibrium shifts in which expression of canonically spliced mRNA (product 15) and aberrantly spliced mRNA (products 16 and 17) are at similar levels. This results in an increase in the expression of the canonically spliced c.1256A>T allele, which has been quantified to be 2.3 fold (Fig 4G). The second allele with the c.1551+1G>T variant is responsible for the majority of canonically spliced exon 8 mRNA. However, in this mRNA, exon 10 is skipped resulting in translation of inactive GAA protein. Without AON exon 8 treatment, the c.1551+1G>T allele is the dominant allele that is preferably amplified in the flanking exon PCR of exon 8. Treatment with AON exon 8 changes the equilibrium of exon 8 splicing, but this has no functional consequences on the c.1551+1G>T allele as its product is nonfunctional in all cases. **(E)** Sequence analysis of products 15-17 identified in **(D)**.

Supplementary Table 1. The number of clones is indicated.

Product Nr.	Product type	- AON	+ AON
13	Retention intron 10	1	1
14	Utilization cryptic c.1552-30	9	3
15	Utilization cryptic c.1537 and c.1552-30	5	5
16	Canonical splicing	23	37
17	Skip exon 10 and use of cryptic c.1552-30	9	-
	total	47	46

Supplementary Table 2. gDNA: genomic DNA, SDM: Site Directed Mutagenesis.

Primer name	Sequence 5' -> 3'	purpose
GAA intron15 gDNA fw	AGGAAACAGGACAGGGCAGAG	gDNA sequencing
GAA intron15 gDNA rv	CTTGGAGGACTCAGGTCAGACG	gDNA sequencing
Minigene *GAA* ex15-17 fw	GCGCCTCGAGCCCAGGAGCCGTACAGCTT	minigene
Minigene *GAA* ex15-17 rv	GCGCGAATTCCTGCAGGGGGATGATGTACC	minigene
GAA c.2190-345A>G fw	TCGTACTTTCCAGATACTTGACTGATGAGC	SDM
GAA c.2190-345A>G rv	CATCAGTCAAGTATCTGGAAAGTACGAAAA	SDM
GAA exon 15-17 minigene fw	GAGCCGTACAGCTTCAGCGA	RT-PCR
GAA exon 15-17minigene rv	TAGAAGGCACAGTCGAGG	RT-PCR
GAA Exon 15-17 fw	GAGCCGTACAGCTTCAGCGA	RT-PCR
GAA Exon 15-17 rv	ATGTACCCAGCCCGGAGGT	RT-PCR
CyPA Exon 1-5 fw	CACCGTGTTCTTCGACATTG	RT-PCR
CyPA Exon 1-5 rv	CCATGGCCTCCACAATATTC	RT-PCR
CyPA Exon 4-5 fw	GGACCCAACACAAATGGTTC	qPCR
CyPA Exon 4-5 rv	GGCCTCCACAATATTCATGC	qPCR
GAA Exon 1-2 fw	AAACTGAGGCACGGAGCG	qPCR
GAA Exon 1-2 rv	GAGTGCAGCGGTTGCCAA	qPCR
GAA exon15mut fw	CAGCAGGCCATGAGGAAG	qPCR
GAA exon15mut rv	GCATGTGCTCATCAGTCAAGTAT	qPCR
GAA Exon 10 fw	GATCCTGCCATCAGCAGCT	RT-PCR
GAA Exon 10 rv	TGGGTTCTCCAGCTCATTGT	RT-PCR
GAA Exon 20 fw	GGGCGGAGTGTGTTAGTCTC	qPCR
GAA Exon 20 rv	CTCCAGGTGACACATGCAAC	qPCR
GAA exon8mut fw	GGCGCTACATGATGATCGTG	qPCR
GAA exon8mut rv	AGTGGACCCGGGCCATAC	qPCR
GAA Exon 9 fw	GACGTCCAGTGGAACGACCT	RT-PCR
GAA Exon 9 rv	ACCTGGTCATGGAACTCAGC	RT-PCR
Neomycin fw	TCATCTCACCTTGCTCCTGC	qPCR
Neomycin rv	GTGGTCGAATGGGCAGGTAG	qPCR
